# First records of *Xyleborinus octiesdentatus* (Murayama) (Coleoptera, Curculionidae, Scolytinae) from North America

**DOI:** 10.3897/zookeys.56.528

**Published:** 2010-09-17

**Authors:** Robert J. Rabaglia, Miloš Knížek, Wood Johnson

**Affiliations:** 1 USDA Forest Service, 1601 N. Kent St, RPC–7, Arlington, VA 22209, USA; 2 Forestry and Game Management Research Institute, Jíloviště - Strnady, CZ-156 04 Praha 5 - Zbraslav, Czech Republic; 3 USDA Forest Service, 2500 Shreveport Hwy, Pineville, LA 71360, USA

**Keywords:** Coleoptera, Curculionidae, Scolytidae, Xyleborina, Xyleborinus octiesdentatus, new North American record, exotic species

## Abstract

Xyleborinus octiesdentatus (Murayama), an ambrosia beetle native to Asia, is reported for the first time in North America based on specimens from Alabama and Louisiana. This is the twenty-first species of exotic Xyleborina documented in North America. A re-description of the female and a key to the four North American species of Xyleborinus are presented.

## Introduction

In 1977, Steve Wood documented the introduced and exported North and Central American scolytines known at that time (Wood 1977). He also pointed out that many of the recent introductions into North America were species with sib-mating systems (Wood 1982). Since then there have been many papers citing the numerous species and increasing rate of introductions of non-native scolytines into North America. The number of non-native scolytines known to be established in North America has more than doubled since 1977. Whereas more than half of the species reported prior to 1977 were in the twig and seed infesting genera Hypothenemus and Coccotrypes, most of the recently established species are ambrosia beetles (Hoebeke and Rabaglia 2008, Rabaglia et al. 2009).

Ambrosia beetles in the Xyleborina are commonly transported in dunnage and crating, and readily establish in new environments due to their cryptic nature and sib-mating behavior (Wood 1982, Atkinson et al. 1990, Brockerhoff et al. 2006). Since 1980, there have been 13 non-native xyleborines found to be established in North America. As of 2009, there were 42 species of Xyleborina known from north of Mexico, and 21 of these are non-native (Rabaglia et al. 2006; Hoebeke and Rabaglia 2008; Rabaglia et al. 2009; Okins and Thomas 2010). This paper reports the first occurrence of the 22nd non-native xyleborine, Xyleborinus octiesdentatus (Murayama), documented from North America, provides a key and description for its identification, and presents recent collection and taxonomic data.

The USDA Forest Service has been pilot testing and later implementing on an operational basis, an early detection and rapid response (EDRR) project for bark and ambrosia beetles since 2001 (Rabaglia et al. 2008). This project utilizes Lindgren funnel traps (Lindgren 1983) and lures which target non-native scolytines that are at high risk of introduction into the United States. As part of this project, in 2008 a study was conducted on the Kisatchie National Forest near Winnfield, Louisiana to determine the effects of lure release rates on beetle response. On April 24, 2008 a sample containing an unidentified xyleborine ambrosia beetle was collected, and images were sent to RJR. The beetle was recognized as a new, non-native species and images were forwarded to MK who suggested it was identical with Xyleborinus octiesdentatus (Murayama, 1931). The identification was then confirmed by RJR after comparison with Murayama’s type specimen of at the US National Museum. This was the first record of this species in North America. An additional specimen was collected on August 14, 2008 in Vance, Alabama also as part of the EDRR project. In 2009, WJ collected additional specimens on the Kisatchie National Forest as part of a delimiting survey in Louisiana. Further collection details are in the Distribution section below.

## Systematics

The Xyleborina (Xyleborini sensu Wood & Bright, 1992) is a large and complex group containing more than 1,200 species. Until Wood’s (1986) reclassification, most species in the tribe were placed in the polyphyletic genus Xyleborus. Recent molecular (Jordal 2002) and morphological (Hulcr et al. 2007) methods have helped create a more stable classification (Hulcr and Cognato 2009). The genus Xyleborinus was established by Reitter (1913), and has at times been considered a synonym of Xyleborus. Since Wood (1986), it has been recognized as a distinct genus. The main character used to distinguish the two genera is the somewhat hidden, conical scutellum in Xyleborinus and the visible, flat scutellum in Xyleborus.

Most publications on Xyleborinus octiesdentatus, including the original description from Korea by Murayama (1931), placed it in Xyleborus (Murayama 1934, Yin et al. 1984, Nobuchi 1985, Wood and Bright 1992). A study by R. A. Beaver (pers. comm.) and MK of Murayama’s type collection at the US National Museum in 2003 indicated that the species should be placed in Xyleborinus (Beaver et al. 2008)

### Xyleborinus octiesdentatus

(Murayama, 1931: 46)

Figs 1 2 3 

#### Diagnosis.

Specimens of Xyleborinus octiesdentatus can be easily distinguished from other members of Xyleborinus occurring in North America by the sulcate shape of the elytral declivity, and the elevated lateral declivital margins which bear four pairs of long, narrow, sharply pointed spines, increasing in length approaching the posterior margin.

#### Revised Key to Xyleborinus in America north of Mexico.

The following key, modified from the key to Xyleborinus in Rabaglia et al. (2006), will enable the identification of the five species of Xyleborinus known from America north of Mexico. It includes the recently established Xyleborinus andrewsi in Florida (Okins and Thomas 2010).

**Table:** 

1	Posterior margin of elytra strongly convergent	Xyleborinus andrewsi (Blandford)
–	Posterior margin of elytra broadly rounded	2
2(1)	Declivital interstriae 1 with small granules, 1 and 3 equally, weakly elevated	3
–	Declivital interstriae 1 without granules and not elevated	4
3(2)	Granules on declivital interstriae 1 and 3 larger, those on 3 pointed, spine-like, slightly incurved; granules on ventrolateral area large, sharply pointed, spine-like, curved slightly downwards and to the suture; declivital interstriae 2 flattened; 2.5–2.8 mm	Xyleborinus alni (Niisima)
–	Granules on declivital interstriae 1 and 3 smaller, obtusely pointed; granules on ventrolateral areas small, less pointed; declivital interstriae 2 slightly impressed; 2.0–2.4 mm	Xyleborinus saxesenii (Ratzeburg)
4(2)	Declivity flattened, declivital interstriae 3 slightly elevated with 3 pairs of small denticles, the pair near the posterior margin largest and often blunt; 1.6–1.9 mm	Xyleborinus gracilis Eichhoff
–	Declivity sulcate, declivital interstriae 3 strongly elevated with 4 pairs of long, narrow, pointed spines increasing in size approaching posterior margin, 2.1–2.4 mm	Xyleborinus octiesdentatus Murayama

**Figure 1.**
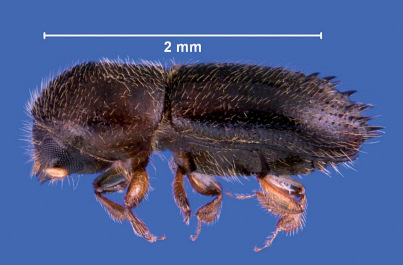
Lateral view of Xyleborinus octiesdentatus adult female.

**Figure 2.**
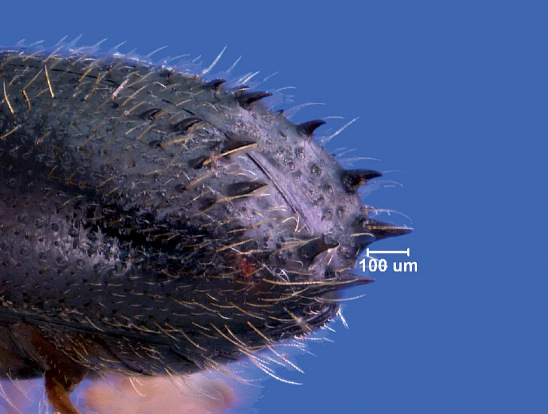
Declivity of Xyleborinus octiesdentatus adult female.

**Figure 3.**
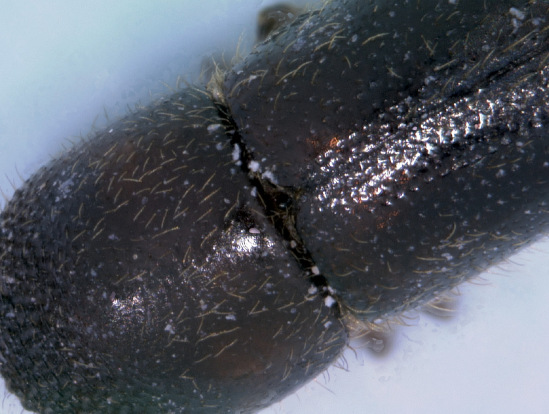
Scutellum of Xyleborinus octiesdentatus adult female.

#### Description.

The species was redescribed by Nunberg (1982)

##### Female.

Length 2.1–2.4 mm (from anterior margin of pronotum to posterior margin of elytra, excluding spines), 2.5× as long as wide; color brown to dark brown, matt. Frons convex, strongly reticulate, with large, shallow, rather dense punctures. Antennal club round with two round sutures on its anterior side and one strongly apically displaced suture on posterior side. Pronotum cylindrical, 1.2× as long as wide, frontal edge broadly rounded bearing small tubercles as continuation of very fine asperities on anterior half, posterior half finely shagreened, with minute, shallow punctures. Scutellum typical for the genus, conical, visible between the emarginated bases of the elytra. Elytra cylindrical 1.5× as long as wide, 1.3× as long as pronotum, side straight on anterior half, narrowed on posterior half; striae on the disk very shallowly impressed, regularly and rather densely punctured; interstriae flat, finely and regularly punctured; interstriae 1, 2 and 3 with small tubercles just before the upper margin of the declivity. Elytral declivity long, nearly from the middle of elytra, sulcate, sutural interstriae weakly elevated on upper part, not elevated below, without granules; lateral edges formed by strongly elevated continuation of 3rd interstriae, which bears four pairs of long, narrow, posteriorly directed sharply pointed spines, which increase in length approaching posterior margin, the last two pair exceeding outline of the elytral edge; several small, fine spines on the lateral raised margin of declivity. Vestiture consisting of moderately long, fine setae in uniseriate rows on interstriae, longer on declivity. Legs yellowish, brown.

##### Male.

Not examined.

#### Distribution.

China, Japan, South Korea (Wood and Bright 1992). United States (all records new to North America, numbers of specimens collected at each location shown in parentheses.): ALABAMA, Tuscaloosa County: Vance, 14 August 2008, ethanol-baited funnel trap (1);LOUISIANA, Winn Parish: Kisatchie National Forest, 6 miles w. of Winnfield, 24 April 2008, α-β-pinene (70:30) and ethanol-baited funnel trap (1); 23 March 2009, α-β-pinene (70:30) and ethanol-baited funnel trap (3), ethanol-baited funnel trap (3), phoebe oil-baited funnel trap (2) ; 9 April 2009, α-β-pinene (70:30) and ethanol-baited funnel trap (1),: ethanol-baited funnel trap (2); 21 April 2009, α-β-pinene (70:30) and ethanol-baited funnel trap (7), ethanol-baited funnel trap (4); 29 April 2009, α-β-pinene (70:30) and ethanol-baited funnel trap (3), ethanol-baited funnel trap (6),: phoebe oil-baited funnel trap (2); 21 May 2009,: α-β-pinene (70:30) and ethanol-baited funnel trap (1); 3 June 2009, trap on girdled yaupon (14); 9 June 2009,: trap on girdled yaupon (3); 15 June 2009, trap on girdled yaupon (3); 14 July 2009, trap on girdled yaupon (2); 4 August 2009,: trap on girdled yaupon (2). All specimens collected in North America are females.

#### Biology, habits, and host.

In its native Asian range, the known hosts for Xyleborinus octiesdentatus are Carpinus laxiflora (Siebold and Zuccarini) Blume (Corylaceae), Cleyera sp., Eurya japonica Thunberg (Theaceae) and Illicium religiosum Siebold (Illiaceae) (Wood and Bright 1992), and Illex rotunda Thunberg (Aquifoliaceae) (Murayama 1934).

Although this species is certainly established in the Winnfield area of Louisiana (Winn Parish) and probably in Alabama (Tuscaloosa Co.), it has not been collected from any host trees. In May, 2009, on the Kisatchie National Forest, Ilex vomitoria Aiton (yaupon), Ilex opaca Aiton (American holly) (Aquifoliaceae) and Carpinus caroliniana Walter (ironwood) (Corylaceae) were girdled and a sticky band placed on the trees to act as attractant/trap trees for the beetle. These species were selected because known hosts of the beetles belonging to these genera occur in Asia. During the month following this treatment 27 specimens were collected on the sticky band on the girdled yaupon whereas no specimens were collected from the other species. No specimens of Xyleborinus octiesdentatus were found boring into the yaupon tree.

The impact Xyleborinus octiesdentatus will have in North America is still uncertain. All species of xyleborines carry symbiotic ambrosia fungi that are usually benign to hosts in their native range. However, as recently discovered with Xyleborus glabratus Eichhoff, 1877, another ambrosia beetle introduced from Asia, certain ambrosial fungi may prove to be very pathogenic on new, novel hosts in North America (Fraedrich et al. 2008). Future studies with Xyleborinus octiesdentatus will attempt to identify fungal associates, and test their pathogenicity against North American host trees.

## Supplementary Material

XML Treatment for Xyleborinus octiesdentatus

## References

[B1] Atkinson TH Rabaglia RJ Bright DE (1990) Newly detected exotic species of Xyleborus (Coleoptera: Scolytidae) with a revised key to species in eastern North America. Canadian Entomologist 122:93-104

[B2] Beaver RA Kajimura H Goto H (2008) Taxonomic changes and new records of Japanese bark and ambrosia beetles (Coleoptera: Curculionidae: Scolytinae). Elytra, Tokyo 36 (2):231-239

[B3] Brockerhoff EG Bain J Kimberley JM Knižek M (2006) Interception frequency of bark and ambrosia beetles (Coleoptera: Scolytinae) and relationship with establishment in New Zealand and worldwide. Canadian Journal of Forest Research 36:289-298

[B4] Fraedrich SW Harrington TC Rabaglia RJ Ulyshen MD Mayfield III AE Hanula JL Eickwort JM Miller DR (2008) A fungal symbiont of the redbay ambrosia beetle causes wilt in redbay and other Lauraceae in the southeastern USA. Plant Disease 92:215-224 10.1094/PDIS-92-2-0215 30769391

[B5] Hoebeke ER Rabaglia RJ (2008) Xyleborus seriatus Blandford (Coleoptera: Curculionidae: Scolytinae), an Asian ambrosia beetle new to North America. Proceedings of the Entomological Society of Washington 110 (2):470-476

[B6] Jordal BH (2002) Elongation Factor 1 alpha resolves the monophyly of the haplodiploid ambrosia beetles Xyleborini (Coleoptera: Curculionidae). Insect Molecular Biology 11:453-465 12230544 10.1046/j.1365-2583.2002.00354.x

[B7] Hulcr J Dole SA Beaver RA Cognato AI (2007) Cladistic review of taxonomic characters in Xyleborina (Coleoptera: Curculionidae: Scolytinae). Systematic Entomology 32 (3):568-584

[B8] Hulcr J Cognato AI (2009) Three new genera of oriental Xyleborina (Coleoptera: Curculionidae: Scolytinae). Zootaxa 2204:19-36 10.11646/zootaxa.4722.6.2 32230598

[B9] Lindgren BS (1983) A multiple funnel trap for scolytid beetles (Coleoptera). Canadian Entomologist 115 (3):299-302

[B10] Murayama J (1931) Révision des familles des Ipides et Platypides (Coléoptères) de l’île de Quelpart. Annot Zool Japonenses 13:39-61

[B11] Murayama J (1934) Notes on the Ipidae (Coleoptera) from Kyushu. Annual Zool Japonenses 14:287-300

[B12] Nobuchi A (1985) Check-list of Coleoptera of Japan. Family Scolytidae. Studies on Scolytidae XXVI. The Coleopterists’ Association of Japan, Tokyo, 32 pp.

[B13] Nunberg M (1982) Die Gattung Xyleborus Eichhoff (Coleoptera, Scolytidae). Erganzungen, Berichitigungen und Erwieterungen der Diagnosen, V. Teil. Annales Zoologici Warszawa. 36: 425–446

[B14] Okins KE Thomas MC (2010) A new North American record for Xyleborinus andrewesi (Coleoptera: Curculionidae: Scolytinae). Florida Entomologist 93 (1):133-134

[B15] Rabaglia RJ Dole SA Cognato AI (2006) Review of American Xyleborina (Coleoptera: Curculionidae: Scolytinae) occurring north of Mexico, with an illustrated key. Annals of the Entomological Society of America 99 (6):1034-1056

[B16] Rabaglia RJ Duerr D Acciavatti R Ragenovich I (2008) Early detection and rapid response for non-native bar and ambrosia beetles. USDA Forest Service, Forest Heath Protection, Washington DC 12 pp.

[B17] Rabaglia RJ Vandenberg NJ Acciavatti, RE (2009) First records of Anisandrus maiche Stark (Coleoptera: Curculionidae: Scolytinae) in North America. Zootaxa 2137:23-28

[B18] Reitter E (1913) Bestimmungs-tabelle der Borkenkäfer (Scolytidae) aus Europa und den angrenzenden Ländern. Wiener Entomologische Zeitung 32:1-116

[B19] Wood SL (1977) Introduced and exported American Scolytidae (Coleoptera). Great Basin Naturalist 37 (1):67-74

[B20] Wood SL (1982) Bark and Ambrosia Beetles of North and Central America (Coleoptera: Scolytidae), a taxonomic monograph. Great basin Naturalist Memoirs No. 6, Brigham Young University, Provo, Utah, 1359 pp.

[B21] Wood SL (1986) A reclassification of the genera of Scolytidae (Coleoptera). Great basin Naturalist Memoirs No. 10, Brigham Young University, Provo, Utah, 126 pp.

[B22] Wood SL Bright Jr. DE (1992) A catalog of Scolytidae and Platypodidae (Coleoptera), part 2: Taxonomic index, Volume A. Great basin Naturalist Memoirs No. 13, Brigham Young University, Provo, Utah, 833 pp.

[B23] Yin HF Huang FS Li ZL (1984) Economic insect fauna of China. Fasc. 29. Coleoptera: Scolytidae. Science Press, Beijing, x + 205 pp., XVII Tab.

